# Can immersive virtual reality be an alternative world for eliciting consumer willingness to pay for food?

**DOI:** 10.1371/journal.pone.0361063

**Published:** 2026-10-22

**Authors:** Toru Nakajima, Mizuki Mukai

**Affiliations:** 1 Department of Data Science for Food Systems, Toyo University, Asaka, Saitama, Japan; 2 Department of Sustainable Resource Sciences, Mie University, Tsu, Mie, Japan; University of Florida, UNITED STATES OF AMERICA

## Abstract

This study examines whether consumers’ willingness to pay (WTP) for ready-to-eat cereals differs between immersive virtual reality (IVR) and real-world (RW) environments. Real choice experiments were conducted with 227 respondents who selected reservation dates without knowing whether they corresponded to the IVR or RW setting. To derive consumers’ WTP, random parameter logit models were estimated primarily in WTP space, while preference-space models were used as a robustness check. The results show no significant differences in WTP between IVR and RW for any of the cereal products tested. This suggests that consumers’ product choices in IVR environments are based on monetary valuations, similar to those in real environments. When combined with real economic incentives, IVR-based choice experiments may therefore yield WTP estimates comparable to those obtained from conventional real choice experiments. To our knowledge, this is the first study to compare WTP between IVR and RW environments using an incentive-compatible experimental design.

## Introduction

Consumers’ food choices are influenced by various factors. To clarify the determinants of food choices, researchers have created substitute environments that mimic real-life settings in multiple ways and conducted experiments and surveys [1]. Recently, research using virtual reality (VR) technology has advanced in different fields, and its applications are expanding in the food industry. According to Xi & Hamari [2], approximately 30% of studies use VR to clarify consumer behavior in food-focused shopping. VR is an effective tool for eliciting consumer behavior toward food [3]. Experiments on food-choice behavior using VR enable virtual recreation of food retail environments, enabling a variety of inexpensive and quick experimental interventions in a confidential setting, without the need for cooperation from retail stores or being affected by external factors [4].

Earlier studies using VR on consumer food-choice behaviors include Waterlander et al. [5], Waterlander et al. [6], Herpen et al. [7], and Bach et al. [8]. Waterlander et al. [5] used randomized controlled trials to analyze how price increases in sugar-sweetened beverages affect the purchase of drinks and snacks in 3D virtual supermarkets. Waterlander et al. [6] examined the effects of a 25% discount on fruits and vegetables on food purchases using web-based 3D virtual supermarkets. Herpen et al. [7] showed that consumer behavior in a 3D environment resembles real in-store behavior more closely than it does in a 2D environment. Bach et al. [8] analyzed whether emphasizing conditions in livestock barns would increase sales of meat products meeting higher animal welfare standards. However, in these studies, VR refers to desktop VR (DVR), in which participants view a PC monitor and use a keyboard or mouse to navigate the 3D space and select products. This type of VR has low immersion [2,3].

Recently, the development and sale of head-mounted displays (HMDs) have enabled immersive VR (IVR) environments, in which the entire 360-degree field of view becomes a virtual space. Recent studies have used IVR environments to elucidate consumer food-choice behaviors. Xu et al. [9] observed behaviors in virtual supermarkets and analyzed the impact of information about animal welfare on meat choice. Melendrez-Ruiz et al. [10] explored the relationship between eye movements and food choice. In all these studies, researchers constructed a virtual supermarket space, replicated it in an IVR environment, and conducted experiments in which participants selected products from shelves within that virtual environment.

Research on food-choice behavior using such IVR environments may help observe behaviors that are closer to those in the real world (RW) and the benefits of conventional VR, given the high level of immersion. Conversely, there are issues such as discomfort caused by wearing an HMD, need to become accustomed to operating the controllers, and possibility of VR sickness [2]. Flash et al. [11] examined the feasibility of experimentally understanding consumer behavior in a virtual supermarket using IVR and found that participants could use the controllers effectively to act within the VR space, mistakes in operating the controllers decreased with training, VR sickness was minimized, and a moderate sense of presence was achieved. These results suggest that interventions in IVR environments safely and effectively clarify consumers’ everyday consumption behavior and can provide a controlled and reliable environment.

Lately, studies comparing consumers’ food-choice behaviors in different environments have been actively conducted, and investigations into the comparability of these environments are progressing. Peukert et al. [12] compared IVR and DVR environments, stating that when selecting muesli products, whether participants wanted to use the same selection environment again did not differ between the two environments. Meißner et al. [13] conducted a real choice experiment (RCE) in DVR and IVR environments, in which RCE is a choice experiment with real payments (details provided in a later section), finding that more muesli products were chosen in the IVR setting and price sensitivity was lower (resulting in higher purchase prices). However, there were no significant differences in satisfaction with product selection between the environments. Yang et al. [14] compared IVR with an online shopping environment and showed that participants experienced significantly more positive emotions after choosing cookies in the IVR environment. This is explained by the higher level of immersion and realism in the IVR environment, which enhances emotional responses, and by the fact that hedonic foods, such as cookies, more easily evoke positive emotions.

Studies comparing IVR environments with RW environments include Pizzi et al. [15], who compared choice behaviors for bakery products. Although they found no significant difference in the quantity of products purchased, participants took longer to select products in the IVR environment, and their satisfaction with the store was lower. This difference in satisfaction narrowed when participants perceived a store’s product assortment as good. Similarly, Siegrist et al. [16] found that when choosing cereals for children, the time spent selecting products was longer in an IVR environment; however, there was no significant difference in the time spent viewing product shelves or in the number of people looking at product information. Furthermore, when participants selected cereals for a friend on a diet, there were no significant differences in the time required for product selection, shelf-viewing time, or the number of people viewing product information. Additionally, Branca et al. [17], who analyzed consumer evaluations of milk packaging, indicated no significant differences in environments, evaluating perceived sustainability or WTP for eco-friendly products. Similarly, Jacobsen et al. [18], who examined consumers’ price memory for pizza sauce, pasta, and dark chocolate, found no significant differences between environments in which consumers could accurately recall sales prices. These studies suggest that IVR environments provide an efficient setting for testing sensory and nonphysical elements and serve as a reliable research tool that reflects realistic consumer behavior. Moreover, Schnack et al. [19] revealed consumer selection behaviors across multiple product categories within IVR environments and compared their findings with those of previous research, indicating that impulse purchasing and information acquisition behaviors in IVR align with reality. Based on these results, consumers also exhibit realistic purchasing behavior in IVR settings; therefore, IVR is a cost-effective alternative method for measuring consumer behavior. Lombart et al. [20] compared fruit and vegetable selection behaviors across three environments: DVR, IVR, and RW. They found that the quantity purchased was higher in DVR and IVR stores than in real stores; however, price appropriateness did not differ significantly between IVR and reality. Further, there were no significant differences among the environments regarding the appearance, quality, and perception of fruits and vegetables as healthy, as well as favorable attitudes toward the products and purchase intentions. Hence, previous research comparing food-choice behaviors in IVR and other environments suggests that behavior in the IVR environment can reproduce actual behavior to a considerable extent, indicating that the IVR environment is an effective method for virtually observing real consumer behavior.

No difference was noted between the IVR and RW environments regarding the time spent viewing product shelves and number of people looking at product information [16], product information acquisition behavior [19], or price recall [18], as well as the perception of appearance and quality [20]. These observations suggest that consumers act according to similar choice mechanisms in similar food selection environments in both settings. This implies that consumers have similar food demands in IVR environments as those in RW. WTP [17] and purchase intentions [20] are also similar in both environments. However, extremely limited studies reveal consumers’ WTP for food products in IVR environments or compare the WTP between IVR and RW settings. Branca et al. [17] examined the WTP for eco-friendly milk packaging compared with conventional milk packaging in an IVR environment and found no significant difference in WTP between the IVR and RW settings. However, in this case, the WTP was based on a seven-point Likert scale and was not derived as a monetary value. Moreover, although the same study conducted a choice-based conjoint analysis in an IVR environment, the monetary value of the WTP was not clarified. Conversely, Meißner et al. [13], who conducted a choice-based conjoint analysis for muesli in an IVR environment, compared the actual payment amount to that in a DVR environment but did not clarify the WTP. Thus, although research on food-choice behavior in IVR environments is progressing, studies that clarify WTP remain in their developmental stages.

This study aims to contribute to the literature by deriving monetary values of consumers’ WTP based on food choices in an IVR environment and clarifying whether there is a difference compared to the WTP derived in an RW environment. Considering the above-mentioned sufficient evidence indicating that consumer behavior is similar in IVR and RW environments, we hypothesize that there is no significant difference between consumer WTP on food elicited under both IVR and RW environments. In doing so, we focus on ready-to-eat cereals (RTEC).

RTEC products are widely consumed globally and have been frequently examined in previous consumer-choice and virtual-shopping studies. RTEC is also suitable for comparing choices between IVR and RW environments because consumers can evaluate the products primarily from visually available information, including packaging, price, nutritional information, and product composition. Unlike foods whose evaluation strongly depends on temperature, aroma, freshness, or immediate tasting, the principal information used in choosing packaged RTEC can be presented in a relatively consistent manner across the two environments. Furthermore, RTEC products are shelf-stable, relatively affordable, and easy to distribute to participants, making them suitable for an incentive-compatible experiment involving actual purchases. The availability of products with distinct characteristics, such as the presence of dried fruit and reduced carbohydrate content, also enables meaningful variation in consumer preferences within a single product category.

## Materials and methods

### Consumer demand for RTEC

Recent food trends focus on health and convenience preferences [21]. Convenience is as important as taste, health, and price in determining consumer preferences for food [22]. RTEC is highly convenient, affordable, easy to purchase, and is sometimes even adopted in nutritional support programs due to its generally high nutritional value [23]. Children and adolescents who frequently consume RTECs tend to consume more sugar [24]. However, RTEC made with whole grains, which are rich in dietary fiber, can slow sugar absorption and are less likely to lead to weight gain [25]. In addition, RTEC containing low-GI carbohydrates is less likely to contribute to weight gain [25].

Consumer demand research has been conducted against the background of RTEC characteristics. Studies that have estimated demand system models for foods, including RTEC, include those that have calculated the price elasticity of demand for RTECs with different nutritional values using the Almost Ideal Demand System (AIDS) model [26], as well as those that have used the Rotterdam model for estimation [27]. In addition, research that has estimated brand-level demand for RTEC using the BLP model [28] has clarified consumer demand for product attributes such as dietary fiber and sugar content, and derived the market dominance of high-concentration industries [29,30].

Conversely, many studies have recently clarified the demand for RTEC through discrete choice experiments and purchasing experiments targeting consumers. The WTP for RTEC increases as the content of sugar, protein, dietary fiber, and saturated fatty acids increases [31]. Further, nutritional information does not significantly influence WTP. Zizzo et al. [32] conducted a purchasing experiment with actual payments in an online supermarket, revealing that although both 20% and 40% tax rates reduced the demand for RTEC, the amount of reduction was not significantly different. They also stated that the tax amount caused a greater decline in demand, even with the same 20% tax, and taxing unhealthy products increased demand for healthier alternatives. Gustafson [33] analyzed consumer choice behavior data from a hypothetical online food choice experiment and showed that when considering the impact on future health, consumers who selected foods with higher nutritional quality were more likely to use nutrition information and were more apt to consider the impact on future health when exposed to information emphasizing the importance of health. Similar experiments have shown that consumers are more likely to select products they believe are healthy or tasty [34]. Furthermore, regarding experiments conducted in an IVR environment elucidating consumer attitudes and selection behavior, some studies have analyzed eye-tracking data during the selection of 33 RTEC products [16], as well as those focusing on muesli [12,13].

### Survey procedure

A four-day discrete choice experiment and questionnaire survey on the aforementioned RTEC products were conducted at the Toyo University Asaka Campus from November 25–28, 2024. The participants were undergraduate and graduate students of the campus who occasionally consumed RTEC, including granola. Although a wide range of age groups consumes RTEC, the proportion of children and adolescents who eat it is higher than that of adults [35]. Additionally, many prior studies comparing IVR and RW have targeted university students [12–14,16,20], with approximately 70% of their participants being students [36]. In some studies, the majority of data were collected from young people up to their early 30s [17].

In the choice experiment, the first two days were conducted in the RW environment and the last two days were conducted in the IVR environment. Before the experiment, participants were informed about it via email and on-campus digital signage, and they made reservations in advance using a reservation system. The reservation system was implemented considering the experiment and survey content. Two to three participants per 20-minute time slot were appropriate, which also helped prevent more than two or three participants from attending the same time slot. The participants accessed the system themselves and made reservations about the experimental time that suited them. Participants were not informed in advance about the specific dates for the RW and IVR environments, and decided on their experiment date and time solely based on their convenience. Therefore, the assignment of participants to one of the two environments can be considered random.

On the experiment day, participants checked in at the designated experimental venue at a specified place on campus. First, they read a written informed consent form and instruction sheet regarding the experimental and survey procedures. Only those who agreed to participate in the experiment were included. After participating in a choice experiment in either an RW or IVR environment, the participants used their smartphones to answer a questionnaire about their personal attributes and usual food consumption behaviors. After completing the questionnaire, participants purchased one of the products they had chosen during the choice experiment. They were given JPY 1,100 as compensation for their cooperation in the experiment, from which the product purchase price was deducted. Specifically, participants were given a voucher instead of cash after agreeing to the contents. After completing the questionnaire, the difference between JPY 1,100 and the product purchase price was paid to the participants in cash. The product to be purchased was determined by the participant’s actual choice of RTEC brands for a randomly selected choice experiment question. The choice experiment had an outside option; that is, participants can choose a no-purchase alternative for a question. If the participant chose the option to buy nothing from a randomly selected question, they were paid JPY 1,100.

In the questionnaire administered after the choice experiment, participants were asked about their personal attributes, such as age, gender, and income. For income, respondents were asked about the amount they could freely spend in the most recent month, calculated as the total from allowances or earnings minus fixed expenses, such as rent. Additionally, to assess food preferences, questions were asked about whether they valued the colorful presentation of meals, whether they liked sweet foods, and whether they were currently on a diet. Furthermore, to determine the factors prioritized when purchasing RTEC, the participants were asked to select all that applied from three options: whether it contained dried fruit, whether it was low in calories and carbohydrates, and whether it was inexpensive. To consider the influence of food experience on WTP, respondents were asked if they had ever tried the RTEC granola alternatives in the choice experiment.

The research protocol for the series of experiments and surveys described here was submitted in advance to the Ethical Review Board for Humanities and Social Sciences Research, Asaka Campus, Toyo University, and was approved by the board. The approval number is 2024−11, and the approval date is November 22, 2024. The form of consent was not formally obtained because the data were analyzed anonymously.

### Real choice experiments

A discrete choice experiment involves having participants make product selection decisions in hypothetical situations. Consequently, the WTP revealed through these experiments may deviate from consumers’ true WTP [37,38]. Various methods mitigate such hypothetical bias [39–41]. Several studies have been conducted using real choice experiments (RCE), which reduce hypothetical bias by having participants purchase products during the choice experiment [13,37,42–49]. In an RCE, one question is randomly selected from the choice experiment questions, and based on the participant’s selection of that question, an actual product purchase is conducted. Compared with the WTP obtained from hypothetical choice experiments (HCE), which do not involve actual purchases, the WTP derived from RCE is lower. This indicates the presence of a hypothetical bias in HCE [37], or sales forecast accuracy is higher with RCE than with HCE [48]. Thus, the RCE is an incentive-compatible method and is more effective than the other methods in reducing hypothetical bias [41]. In this study, RCE was conducted in both the IVR and RW environments to derive the WTP in both settings.

In the discrete choice experiment, participants were asked to choose one RTEC granola product they wanted to purchase from three types—Mygra, Frugra, and Frugra LC—in nine trials, each with different combinations of product prices. These products were produced by Calbee, Inc., a leading company of granola products. Mygra has no dried fruits while Frugra contains dried fruits such as raisins, strawberries, and apples. Frugra LC is a low-carbo type of Frugra, which contains 30% less sugar than the original Frugra by using soy protein. If they did not wish to buy any, they were instructed to select the “Buy neither” option. In this label-based choice experiment, three price levels were set for each product and nine combinations were selected based on an orthogonal design. The price levels for each product were determined with reference to retail prices at supermarkets near the universities, as shown in Table 1. These nine combinations were the same in both experimental settings, IVR and RW. Since the package volumes differed—Mygra was 700 g, Frugra was 750 g, and Frugra LC was 600 g—the price tags for each product included the price per 100 g, in addition to the base price. All displayed prices followed tax-included pricing, as recommended for product displays in Japan.

**Table 1:** Attribute and its levels in the DCE.

	Mygra	Frugra	Frugra LC
**Price**	550 (78.57) 600 (85.71) 650 (92.86)	650 (86.67) 700 (93.33) 750 (100)	650 (108.33) 700 (116.67) 750 (125)

Notes:

1) Values are prices per bag, including consumption tax. Values in parentheses are prices per 100 g.

### Experimental design

In the RW environment, three types of products and their price tags were placed on real shelves in a laboratory (Fig 1), and nine of these shelves (the same number as in the choice experiments) were set up. Participants marked the products they wanted to buy from each shelf on a checklist. If they did not want to buy any, they marked “Buy neither.” After choosing all nine shelves, the participants answered a questionnaire.

**Fig 1:**
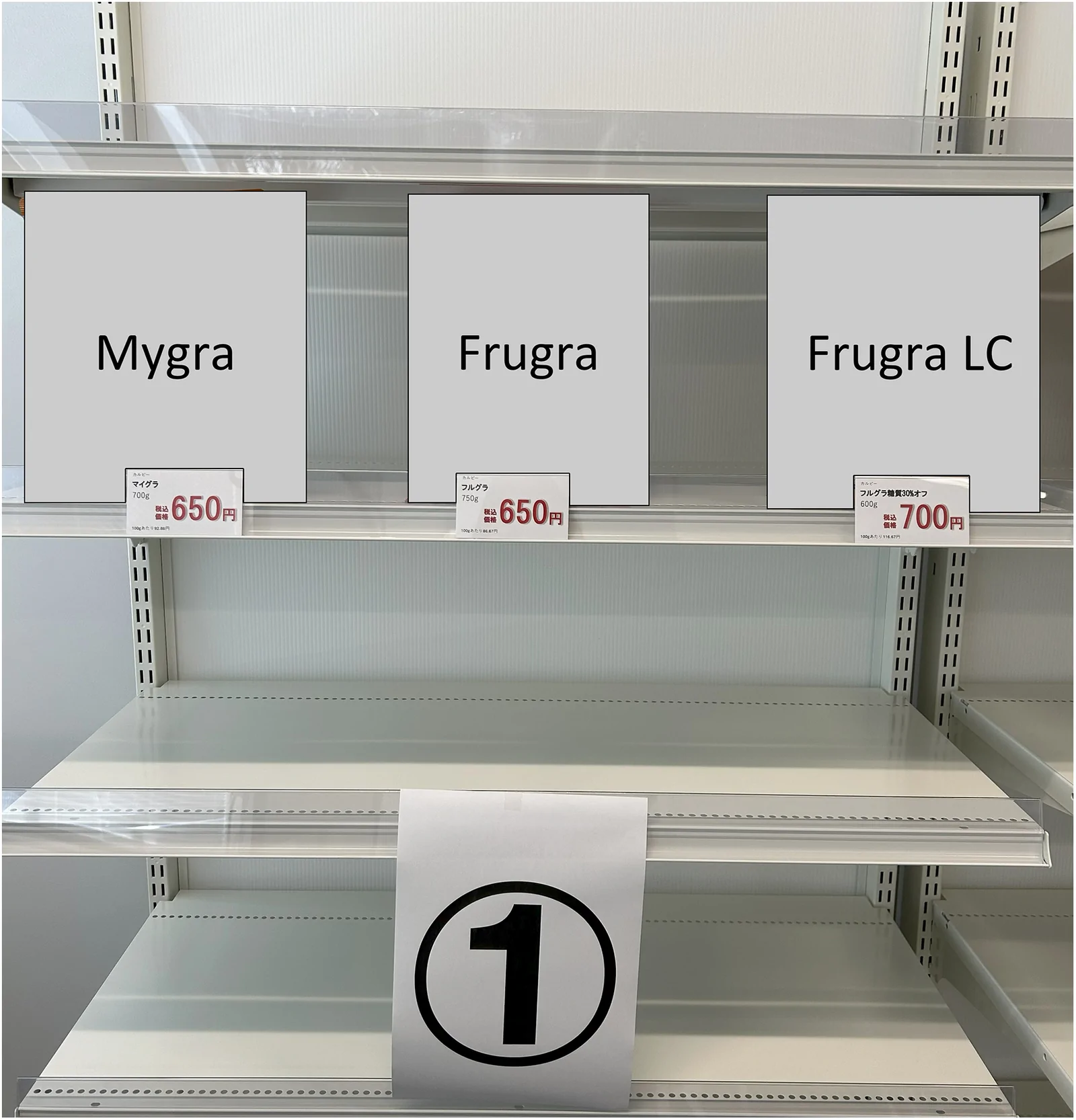
Product shelves in the RW environment. Source: Authors. Notes: 1) Three products were placed in identical order on all nine display shelves. The products are, from left to right, Mygra, Frugra, and Frugra LC. 2) Each price tag displays the name of the product, its weight, and price, including taxes. Each shelf had a different combination of cereal prices, as determined by the experimental design. 3) The photograph was taken by the authors. To avoid reproducing copyrighted package artwork, the original product packages were fully obscured and replaced with author-created schematic representations. All price labels were created by the authors specifically for the experiment.

In the IVR environment, three sets of desks and chairs equipped with HMDs were prepared, allowing up to three participants to participate in the experiment simultaneously (Fig 2). For this experiment, Meta Quest Pro by Meta was used as the HMD. The virtual space used a 3D supermarket environment from the Unity Asset Store, which was modified to allow multiple rounds of selection experiments. When the participants put on the HMD, they found themselves inside a virtual supermarket with a shelf displaying the three RTEC products in front of them. The selection system allowed respondents to use the controller to grab the product they wanted to buy and place it on a shopping cart located to the left of the shelf (Fig 3). If a respondent does not wish to purchase any of the products, they press the “Buy neither” button in the message box above the product shelf or place a black-box product in the lower rows of the shelf into the cart to confirm their selection. All selection results were recorded and reviewed as a CSV file at the end of the experiment. After the session ended, a CSV file with the recorded selection results was printed and used when choosing a product to purchase. In addition, before conducting the nine main selection experiments, three practice rounds were provided, during which participants familiarized themselves with the product selection and operation methods under the experimenter’s guidance. Each HMD was connected to a separate laptop, and the footage viewed through the HMDs by the participants was mirrored on the laptops, allowing the experimenter to easily check both the participants’ actions and what they were seeing. After all nine selection experiments were completed, the participants removed their HMDs and answered a questionnaire.

**Fig 2:**
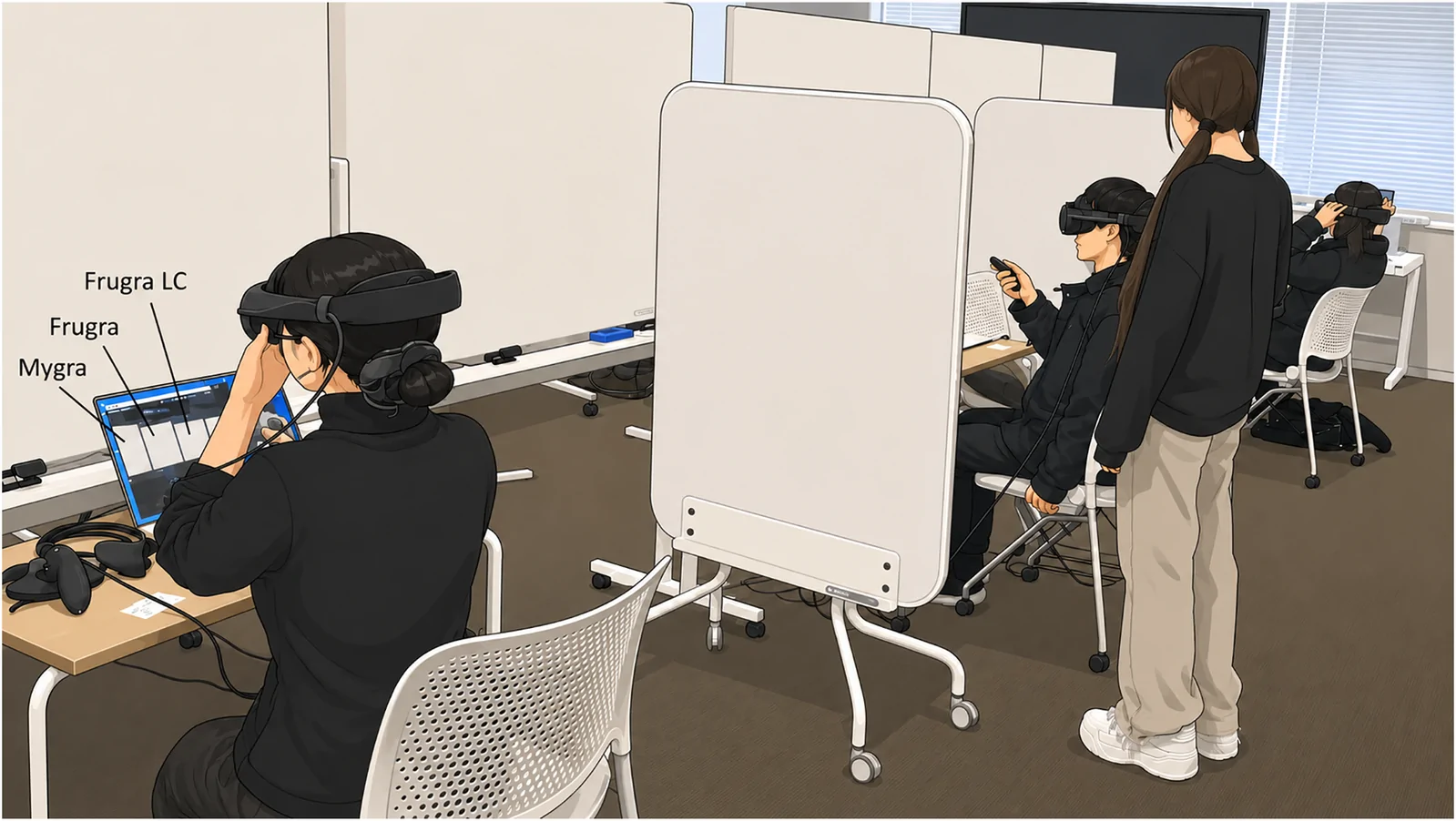
Cereal choice experiments in the IVR environment. Source: Authors. Notes: 1) The experiment was conducted with a maximum of three participants simultaneously. The content shown on the HMD display was made available to the experimental staff via a PC, who supervised whether the participants were taking appropriate selection actions and provided support if the participants had questions on how to operate the HMD. 2) The illustration was created by the authors based on a photograph taken during the experiment. To protect participant privacy, all individuals were rendered in a generalized, non-identifiable form. To avoid reproducing copyrighted package artwork, the artwork on the product packages visible on the monitors was fully obscured and replaced with author-created schematic representations.

**Fig 3:**
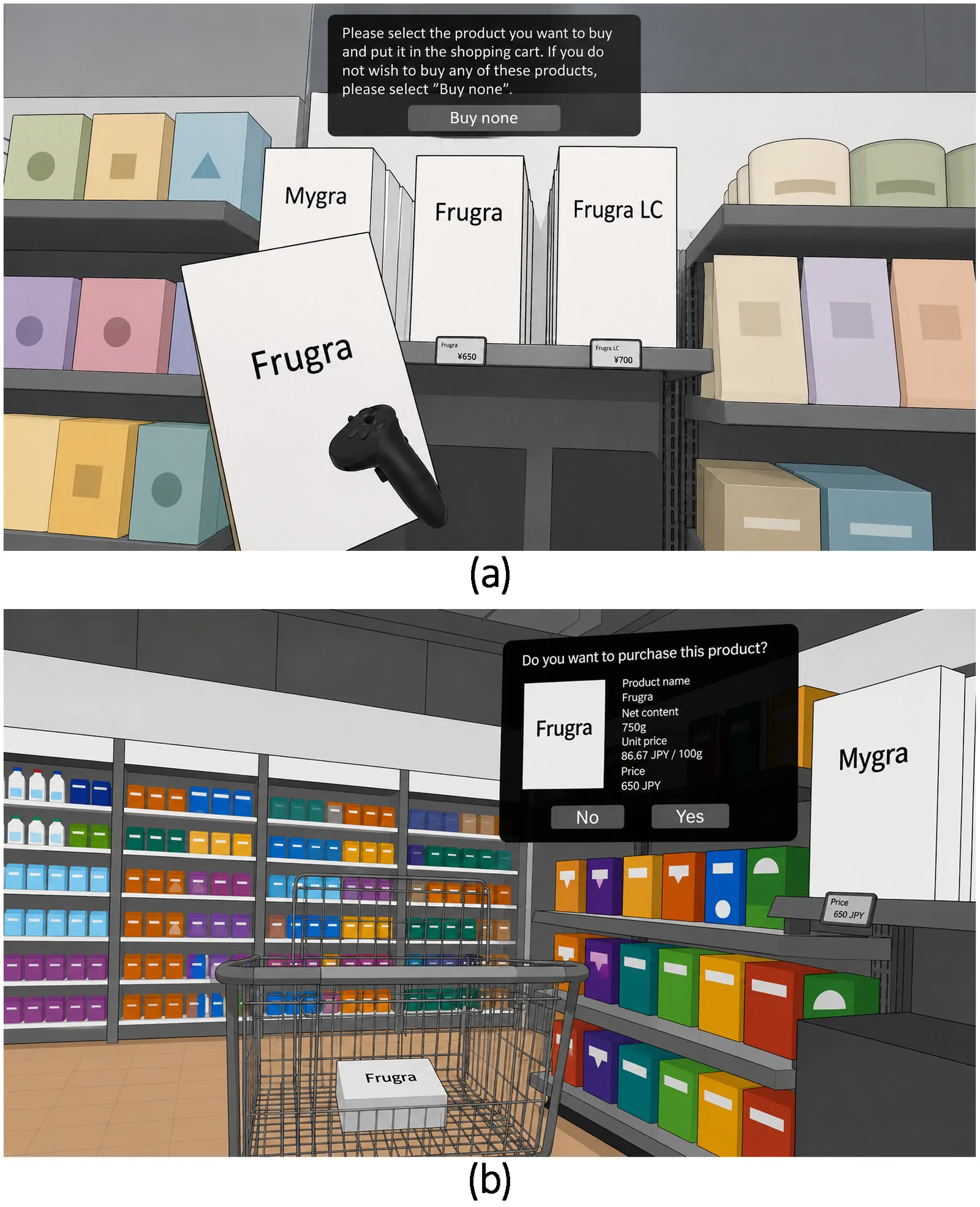
Cereal selection scenes displayed on the HMD. Source: Authors. Notes: 1) The order in which the cereals are displayed and the prices shown are the same as those in the RW environment. 2) Each item can be grabbed by pressing a button on the controller (Fig 3a). When the button is released, the product is released (falls). Three identical products are placed on the shelf for each cereal so that the respondent can immediately select a product if they accidentally release the product that they were grabbing. However, only one product can be selected per question. 3) Once the grabbed product is placed in the cart, a message is displayed asking if the respondent wants to confirm the selection of the product (Fig 3b). 4) If the respondent does not wish to purchase any of the products, they should press the “Buy neither” button in the message box above the product shelf (Fig 3a), or put a black-box product in the lower rows of the shelf into the cart to confirm his/her selection. 5) After completing the specified number of selections (nine questions in this case), the selection data are recorded in a file by exiting the application. 6) The system does not allow the selection of products other than the three cereals. 7) The illustrations were created by the authors based on screenshots captured in the virtual environment. To avoid reproducing copyrighted package artwork, the artwork on the original product packages was fully obscured and replaced with author-created schematic representations. The virtual supermarket environment, including the shelves, store fixtures, and surrounding products, was rendered in a substantially simplified and abstracted form. The original screenshots are not reproduced.

In either environment, participants were free to pick up the products and view the nutrition facts labels, as well as the information on the back of the product package. Scanned images of actual product packages were used in the IVR environment. The nutritional information was positioned at the back of the IVR environment for better visibility. Additionally, although the actual product packages were bagged in plastic bags, as shown in Fig 1, the objects were simplified into box shapes in the IVR environment to improve realism without compromising processing speed [16].

### Analytical methods

Using the data obtained from the choice experiments, we derived the WTP for the three RTEC brands. The WTP can be computed by estimating a discrete choice model based on responses from choice experiments. In this study, we used a discrete choice model based on the random utility theory. According to this theory, individuals’ utility can be divided into explainable and unexplainable components. The indirect utility $U_{nit}$ when consumer n selects product i from choice set t is represented by


$$\begin{array}{c} \begin{array}{c} U_{nit}=V_{nit}+\varepsilon_{nit}=-\alpha_{n}p_{nit}+\beta_{n}^{\prime }x_{nit}+\varepsilon_{nit}. \end{array} \end{array}$$ (1)

Here, $V_{nit}$ is explained by price variables $p_{nit}$ and non-price variables $x_{nit}$, and $\varepsilon_{nit}$ is an IID disturbance term that follows a type 1 extreme value distribution (Gumbel extreme value distribution) with mean 0 and variance $\sigma_{n}^{\text{2}}\left(\frac{\pi^{\text{2}}}{\text{6}}\right)$.

Dividing equation (1) by $\sigma_{n}$ yields


$$\begin{array}{c} \begin{array}{c} \frac{U_{nit}}{\sigma_{n}}=-\frac{\alpha_{n}}{\sigma_{n}}p_{nit}+\frac{\beta_{n}^{\prime }}{\sigma_{n}}x_{nit}+\frac{\varepsilon_{nit}}{\sigma_{n}}. \end{array} \end{array}$$ (2)

The error term $\frac{\varepsilon_{nit}}{\sigma_{n}}$ is distributed as a Gumbel extreme value distribution with mean 0 and variance $\frac{\pi^{\text{2}}}{\text{6}}$. Equation (2) expresses the utility function in the preference space [50].

If we let $\lambda_{n}=\frac{\alpha_{n}}{\sigma_{n}}$ and $w_{n}^{\prime }=\frac{\beta_{n}^{\prime }}{\alpha_{n}}$ in equation (2), then the equation can be expressed as


$$\begin{array}{c} \begin{array}{c} \frac{U_{nit}}{\sigma_{n}}=\lambda_{n}\left(-p_{nit}+w_{n}^{\prime }x_{nit}\right)+\frac{\varepsilon_{nit}}{\sigma_{n}}. \end{array} \end{array}$$ (3)

As $w_{n}^{\prime }$ represents the MWTP, the coefficients of $x_{nit}$ in equation (3) become the MWTP. This expression is called the utility function in the WTP space. $\lambda_{n}$ is called the scale parameter, which is estimated as the coefficient of the price variable.

In a multinomial logit model (conditional logit model), the probability that consumer n selects product i from a set of choices t is expressed as


$$\begin{array}{c} \begin{array}{c} P_{nit}=\frac{\text{exp}V_{nit}}{\sum_{j=\text{1}}^{J}\text{exp}V_{njt}}. \end{array} \end{array}$$ (4)

Meanwhile, in the random parameter logit (RPL) model, the choice probability can be expressed as [51]


$$\begin{array}{c} \begin{array}{c} P_{ni}=\int \left[\prod_{t=\text{1}}^{T}\left(\frac{\text{exp}V_{nit}}{\sum_{j=\text{1}}^{J}\text{exp}V_{njt}}\right)\right]f(\theta )\text{d}\theta . \end{array} \end{array}$$ (5)

Here, θ indicates the parameter vector including α and β, and f(θ) represents the distribution of the random parameters. We employed the RPL model to account for unobserved heterogeneity in consumers’ preferences for the cereal products.

We conducted a labeled choice experiment comprising three types of products and an outside option. As the only product attribute is price, the simplest $V_{nit}$ model includes alternative-specific constants for each product and price as the product attribute (Model 1). A common price parameter was used across the three products in all specifications. Furthermore, because the main objective of this study is to clarify whether the WTP differs between the IVR and RW environments, Model 2 adds interaction terms between each alternative-specific constant and a VR dummy variable (which takes the value 1 in the IVR environment and 0 otherwise) to the variables in Model 1. Additionally, by considering the effect of consumer characteristics such as personal attributes on WTP, we can reveal whether WTP differs between IVR and RW environments when consumer characteristics are held constant. Therefore, Model 3 adds the interaction terms between the alternative-specific constants and consumer characteristic variables to Model 2. For all models, the main analysis was conducted in WTP space because it allows WTP distributions to be specified directly and avoids the instability that may arise when WTP is calculated as a ratio of random parameters in preference space [53]. Meanwhile, the preference-space models were estimated as a robustness check. In the WTP-space estimation, we assumed random parameters for all variables except price, each following a normal distribution. The scale parameter associated with price was assumed to follow a log-normal distribution, ensuring that the marginal utility of price had the theoretically expected negative sign. We used the maximum simulated likelihood to estimate the random parameter logit model. In the simulation for Model 1, random numbers were generated 1,000 times using the Halton sequence. However, in the simulations for Models 2 and 3, because there were random parameters for five or more variables, random numbers were generated 1,000 times using the Sobol sequence [52]. The analyses were conducted using the R logitr package [53].

## Results

### Descriptive statistics

Of the 227 participants, none refused to respond or withdrew during the study. Among the participants assigned to the IVR environment, none reported feeling unwell or making operational errors. Therefore, all 227 participants were considered valid respondents.

Table 2 presents the descriptive statistics results for the VR dummy variables and consumer characteristics. The number of participants was nearly equal between the first and last two days, and 49% (those who took part in the previous two days) were assigned to the treatment group in the IVR environment. Regarding individual attributes, about 60% of the participants were female, the average age was approximately 21 years old, and the average monthly income was about JPY 44,000. In terms of food preferences, only approximately 20% of the participants prioritized colorful meals or reported being on a diet, whereas many responded that they liked sweet meals. As this survey targeted those who eat RTEC and most RTEC products are sweet to some extent, this may have led to a high number of respondents who preferred sweet foods. When asked what they prioritized when purchasing RTEC, approximately half valued the inclusion of dried fruits, whereas less than 30% prioritized low-calorie or low-sugar content. Additionally, as most participants were students, over 70% emphasized the importance of low prices. Regarding food experience, over 60% had eaten Frugra before, indicating high brand recognition as a top brand. Conversely, only about 20% had tried Mygra or Frugra LC, suggesting a lower recognition of these products.

**Table 2:** Descriptive statistics and treatment/control differences.

Variable	Mean	S.D.	Min	Max	Obs.	Diff.
**vr (1 in IVR, 0 in real)**	0.49	0.5	0	1	227	–
**fem (1 if female, 0 otherwise)**	0.59	0.49	0	1	227	n.s.
**age**	20.69	1.74	18	29	227	n.s.
**inc (income per month in 10 thousand JPY)**	4.38	2.87	0.5	15.5	227	n.s.
**col (1 if valuing the colorfulness of the meal, 0 otherwise)**	0.19	0.4	0	1	227	n.s.
**swt (1 if liking sweets, 0 otherwise)**	0.69	0.46	0	1	227	n.s.
**diet (1 if being on a diet, 0 otherwise)**	0.17	0.37	0	1	227	n.s.
**df (1 if preferring RTEC with dried fruits, 0 otherwise)**	0.53	0.5	0	1	227	n.s.
**lc (1 if preferring RTEC with low calories/sugar, 0 otherwise)**	0.27	0.45	0	1	227	n.s.
**lp (1 if preferring lower price RTEC, 0 otherwise)**	0.73	0.44	0	1	227	n.s.
**eatmg (1 if having eaten Mygra, 0 otherwise)**	0.22	0.41	0	1	227	n.s.
**eatfg (1 if having eaten Frugra, 0 otherwise)**	0.66	0.47	0	1	227	n.s.
**eatlc (1 if having eaten Frugra Carbohydrate 30% OFF, 0 otherwise)**	0.20	0.40	0	1	227	n.s.

Notes

1) “n.s.” in “Diff.” indicates no significance at the 10% level between treatment (VR = 1) and control (VR = 0) groups using t-tests for quantitative variables (age and inc) or chi-squared tests for categorical variables.

Table 2 also shows the results of t-tests (for quantitative variables) and chi-square tests (for qualitative variables) conducted to determine whether the mean values (for quantitative variables) or frequencies (for qualitative variables) of each variable differed between the treatment (IVR environment) and control (RW environment) groups. According to these results, it cannot be said that the respondents’ characteristics differ depending on whether they are in the treatment group. Thus, consumer characteristics are considered well-balanced between the groups.

### WTP estimates

Table 3 presents the estimation results of the discrete choice model using the utility function of the WTP space. In the WTP-space estimations, the coefficients of all variables other than the scale parameter directly represent MWTP. According to the results of Model 1, the average WTP per bag is approximately 633 yen for Mygra, 736 yen for Frugra, and 707 yen for Frugra LC, which translates to JPY 90, 98, and 118 per 100 g, respectively. While all these are within the retail price range of nearby stores, they can be considered somewhat high.

**Table 3:** WTP Estimation results.

	Model 1		Model 2		Model 3	
	Mean	SD	Mean	SD	Mean	SD
**Scale parameter**	-3.22***	0.44***	-3.23***	0.42***	-3.17***	0.59***
**ASC_MG**	633.02***	73.27***	631.39***	74.55***	814.50***	51.16***
**ASC_FG**	736.34***	58.75***	740.94***	51.92***	713.81***	10.79**
**ASC_LC**	707.21***	79.90***	717.48***	76.53***	641.86***	24.79***
ASC_MG * vr			-5.01	12.95*	1.45	36.04***
ASC_FG * vr			-7.10	33.41*	-6.19	27.50***
ASC_LC * vr			-12.15	2.60	-18.02	65.23***
ASC_MG * age					-10.65***	0.96***
ASC_FG * age					-1.38	1.76***
ASC_LC * age					-0.18	0.67
ASC_MG * fem					-1.92	0.35
ASC_FG * fem					15.76*	11.48
ASC_LC * fem					42.25***	9.28
ASC_MG * inc					5.49***	4.62***
ASC_FG * inc					3.67*	6.37***
ASC_LC * inc					1.87	7.45***
ASC_LC * diet					1.63	0.08
ASC_LC * swt					3.53	11.69
ASC_FG * col					6.13	24.60**
ASC_LC * col					17.11	12.59
ASC_FG * df					52.25***	16.26**
ASC_LC * df					35.91***	0.02
ASC_LC * lc					56.47***	6.26
ASC_MG * lp					24.96**	29.92***
ASC_MG * eatmg					28.35***	34.36***
ASC_FG * eatfg					7.30	26.20***
ASC_LC * eatlc					52.50***	37.04***
**Number of observations**	2043.00		2043.00		2043.00	
**Log-Likelihood**	-1807.54		-1,809.30		-1,738.44	
**AIC**	3631.078		3,646.59		3,584.88	
**BIC**	3676.056		3,725.30		3,888.48	

Note:

1) All estimation results are based on RPL models specified in WTP space.

2) MG, FG, and LC indicate Mygra, Frugra, and Frugra LC, respectively.

3) Values are MWTP per bag of each RTEC commodity. Because the weights per bag of Mygra, Frugra, and Frugra LC were 700 g, 750 g, and 600 g, respectively, the MWTPs per 100 g were obtained by dividing the values in the table by 7, 7.5, and 6.

4) ***, **, and * indicate significance at the 1%, 5%, and 10% levels, respectively.

In Model 2, the WTP for all three RTEC tended to be lower in the IVR environment than in the RW environment; however, this difference was not statistically significant. The results from Model 3 also showed that the environment had no statistically significant effect on WTP for any of the products. These findings suggest that WTP does not differ between the virtual and real environments. The results remained unchanged when the price coefficient was specified as fixed in both WTP space and preference space; these results are reported in S1 and S2 Tables, respectively. Models assuming a zero-censored normal price coefficient in WTP space or a log-normal or zero-censored normal price coefficient in preference space failed to converge.

Considering the differences between virtual and real environments, the relationship between individual attributes and WTP revealed that for every yearly increase in age, the WTP for Mygra decreased by JPY 10.6 per bag (JPY 1.5 per 100 g). This indicates that Mygra is relatively well accepted among younger students. Women have a higher WTP for Frugra than men by JPY 15.8 per bag (JPY 2.1 per 100 g), and a higher WTP for Frugra LC by JPY 42.3 per bag (JPY 7.0 per 100 g). The results also showed that a JPY 10,000 increase in monthly income was associated with increases in WTP of JPY 5.5 per bag (JPY 0.8 per 100 g) for Mygra and JPY 3.7 per bag (JPY 0.5 per 100 g) for Frugra.

The effects of attitudes toward RTEC and eating experiences on WTP were also derived while controlling for differences between virtual and real environments. People who value lower prices have a higher WTP for Mygra, whereas those who prioritize lower calories or lower sugar content have a higher WTP for Frugra LC. In addition, people who value the inclusion of dried fruits have a higher WTP for Frugra and Frugra LC, both of which contain dried fruit. Regarding the eating experience, the results showed that for Mygra and Frugra LC, those who tried the product had a higher WTP. In contrast, eating experience did not significantly affect the WTP for Frugra.

## Discussion

Based on the analysis results from both the IVR and RW environments, the models did not show significant differences in WTP between environments for any product. Therefore, no difference in WTP was noted between a virtual and a real environment. This result is consistent with those of previous studies [16–19].

The present study adds new evidence to this literature by estimating and comparing monetary WTP between IVR and RW environments using the RCE. Although Branca et al. [17] found no environmental difference in “WTP,” their WTP measure was based on a seven-point scale rather than monetary values. Moreover, Meißner et al. [13] employed a conjoint analysis but did not estimate WTP. The present results therefore show that consumers’ monetary valuations derived from actual purchasing decisions can also be comparable between IVR and RW environments.

One possible explanation for the statistically insignificant difference in WTP between the two environments is that participants processed the product and price information similarly in both environments. Previous studies found no substantial environmental differences in viewing product information [16], recalling food prices [18], acquiring product information [19], or evaluating product appearance, quality, and price appropriateness [20]. Because the RTEC products in this study could also be evaluated mainly from visually available information, such as packaging, prices, ingredients, and nutritional information, the similarities reported in these studies may also apply to the RTEC choices examined here. This may explain why WTP did not differ significantly between the IVR and RW environments.

Previous studies have reported differences between IVR and RW environments in selection time, store satisfaction, and purchase quantity [15,16,20]. However, these outcomes concern the choice process or broader shopping behavior rather than the monetary valuation of individual products and are therefore not directly comparable with the WTP examined in this study. Accordingly, these findings do not contradict the present result that WTP was similar across the two environments; they indicate that IVR and RW environments may differ in behavioral dimensions that were outside the scope of the present analysis.

The finding that WTP for Mygra decreases as age increases suggests that Mygra is more accepted among younger students. Combined with the finding that WTP for Mygra decreased as income declined, this could be because Mygra is a relatively inexpensive product, which likely increases its demand among younger students. Additionally, women have a higher WTP for Frugra and Frugra LC than men, possibly because these products contain dried fruits, which are more popular among women. Furthermore, the greater demand for low-carbohydrate options among women suggests that women are more conscious of carbohydrate content than men. These results are consistent with those of previous studies, indicating that women tend to eat more fruit and follow healthier diets than men [54], as well as studies showing that women generally have a more negative view of sugar [55].

The results showed that people who prioritize low prices when purchasing RTEC have a higher WTP for Mygra, suggesting that Mygra is a cost-effective option for price-sensitive consumers. This finding indicates that those who value low calories and sugar content have a higher WTP for Frugra LC, implying that for those looking to limit calories and sugar, products with lower sugar content are attractive. These results suggest that product features align well with consumer demand. Moreover, people who prioritized the inclusion of dried fruit tended to have a higher WTP for both Frugra and Frugra LC, indicating that dried fruit is an important value-adding element of these products. Furthermore, the finding that those who have previously eaten Mygra or Frugra LC show a higher WTP suggests that first-hand tasting experience plays a significant role in stimulating demand. This aligns with previous research showing that familiar foods are more likely to be preferred and consumed [56,57] and food neophobia—hesitancy or aversion to unfamiliar foods—is linked to reduced willingness to try them among neophobic consumers [58]. However, Frugra is a top brand with high recognition, and because many people (two-thirds of the participants in this experiment) have already tried it, the effect of becoming acquainted with taste on demand can be considered limited.

## Conclusions

This study examined whether consumers’ WTP for RTEC products differs between IVR and RW environments using incentive-compatible choice experiments. No significant differences in WTP were found between the two environments for any of the three products. The results therefore indicate that consumers’ monetary valuations of the packaged food products examined in this study were comparable across IVR and RW settings.

From a theoretical perspective, the findings suggest that changing the choice environment from a physical setting to an immersive virtual one does not necessarily alter the reservation prices underlying consumer choices. In microeconomic theory, the WTP for a single product is referred to as its reservation price, which represents the value on the vertical axis of the demand curve at the corresponding consumption quantity [59: 152–153, 416]. When comparable product and price information is provided, IVR may therefore serve as an alternative environment for eliciting consumers’ monetary valuations of packaged foods such as RTEC.

The findings also suggest that the use of an immersive virtual setting does not necessarily undermine the incentive compatibility of an RCE. Although the sense of realism created by IVR may help participants perceive the choice situation as similar to an actual purchasing environment, immersion and realism alone may not be sufficient to reduce hypothetical bias. In the present study, participants faced real economic consequences because one of their choices was implemented through an actual purchase and payment. Previous studies have shown that such incentive-compatible procedures can reduce hypothetical bias [37,41,48]. Thus, combining IVR with real economic incentives may provide WTP estimates comparable to those obtained in a conventional RCE.

The findings also have implications for food-policy and retail-policy evaluation. IVR-based real choice experiments could provide a controlled setting for the preliminary assessment of interventions such as food taxes, subsidies, nutritional labels, health messages, and changes in product presentation or placement. Because virtual stores can be modified without changing an actual retail environment, they may reduce the financial and logistical burden of testing multiple interventions. By accumulating experimental evidence on how such interventions affect WTP in IVR environments, researchers and policymakers could identify factors associated with changes in consumer demand more efficiently than through experiments conducted in real environments [4]. The present study suggests that IVR may help capture the monetary valuations underlying consumers’ food choices, particularly when comparable product information and real economic incentives are provided. Thus, IVR-based RCEs may serve as a useful preliminary tool for evaluating potential food and retail policies before conducting large-scale field experiments.

This study has several limitations. First, the IVR and RW environments were not identical in all respects. In this study’s IVR environment, after adding the selected product to the cart and proceeding to the next question, the system was designed so that participants could make further product choices in the following questions from the same viewpoint as in the previous question. By contrast, in the RW environment, because the experimental design required participants to make multiple discrete product choices with varying price combinations, multiple product shelves were set up, with participants choosing products from each shelf and checking their choices on a form that documented the number of questions and product names as alternatives. Furthermore, in the IVR environment, a supermarket space was virtually constructed, and in addition to the shelves displaying the target products, other items were also displayed. However, because the shelves with the target products were displayed at the front and items other than the target products could not be chosen, the participants focused solely on selecting the target products. In the RW environment, non-target products were not displayed partly because there was not enough space to display such products and partly because respondents could focus on the experiment. This study aimed to clarify whether consumers make product choices based on similar monetary evaluations in the IVR and RW environments. Therefore, it is not necessary to construct identical environments for IVR and real life. However, if we could investigate the differences in WTP in both environments by minimizing differences other than whether the environment was IVR or real, it would allow us to more rigorously identify the effect of IVR on WTP. While it is possible to replicate only the product shelves displaying the target products in the IVR setting [12,13], it is also important to consider providing a sense of realism so that participants feel as though they are choosing products in a retail store, as this can help reduce hypothetical bias. In fact, differences in realism affect the WTP and certainty of choice [60].

Second, the participants in this study were undergraduate and graduate students, as in several previous studies comparing consumer behavior across virtual and other shopping environments [12–14,16,20]. Therefore, the external validity of the present findings is limited. Nevertheless, there are studies targeting a broad age range, including older adults, in which no significant differences in WTP [17] or other attitudes and behaviors [15,18,19] have been observed. Even when older adult participants were included, participants could use controllers properly in VR environments, interventions in IVR settings were safe and effective for clarifying consumers’ everyday consumption behaviors, and these environments provided a controlled and reliable setting [11]. These findings suggest that the comparability between IVR and RW environments may not be limited to student samples. However, since WTP itself is likely to differ between younger and older consumers, whether the observed similarity in WTP between the IVR and RW environments also applies to non-student consumers remains an empirical question.

Third, the time at which each participant took part in the experiment was not explicitly included in the analysis. Consequently, the possible influence of hunger on product choices and WTP could not be examined. However, experiment time alone would not provide an accurate measure of hunger because hunger may vary depending on the timing and amount of the participant’s last meal, individual eating habits, and activities before the experiment, etc. Thus, even if experiment time had been included in the analysis, individual differences in hunger could not have been fully accounted for.

Future research should compare IVR and RW environments in which differences in store layout, product assortment, and choice procedures are minimized, while maintaining sufficient realism for participants to feel that they are choosing products in a retail store. Studies should also recruit general consumers with more diverse demographic and consumption characteristics to examine whether the similarity in WTP between IVR and RW environments can be generalized beyond the student sample used in this study. In addition, similar experiments should be conducted with other food and non-food products to accumulate evidence on the conditions under which WTP estimates obtained in IVR are comparable to those obtained in RW environments. Future studies should also directly measure participants’ hunger, for example by recording subjective hunger levels and the time elapsed since their last meal. Finally, comparing HCE and RCE within an IVR environment would help clarify how real economic incentives affect hypothetical bias, whereas comparing conventional questionnaire-based DCEs with DCEs conducted in IVR would help identify how immersion and the presentation of a virtual shopping environment affect consumers’ choices and estimated WTP.

## Supporting information

S1 Table WTP Estimation results (fixed price parameters). (DOCX)

S2 Table Discrete choice model estimation and WTP results (preference space). (DOCX)
