# Supplementary material for: Can immersive virtual reality be an alternative world for eliciting consumer willingness to pay for food?
Source: PLoS One. 2026 Oct 22;21(10):e0361063. doi: 10.1371/journal.pone.0361063 (PMC13690231; doi:10.1371/journal.pone.0361063)
Supplement: Supplementary file 1 [file pone.0361063.s001.docx]

## S1 Table. WTP Estimation results (fixed price parameters)

|  | Model 1 | | | | Model 2 | | | | Model 3 | | | |
| --- | --- | --- | --- | --- | --- | --- | --- | --- | --- | --- | --- | --- |
|  | Mean | | SD | | Mean | | SD | | Mean | | SD | |
| Scale parameter | 0.04 | *** |  |  | 0.04 | *** |  |  | 0.04 | *** |  |  |
| ASC_MG | 632.01 | *** | 73.18 | *** | 637.01 | *** | 68.52 | *** | 766.77 | *** | 39.52 | *** |
| ASC_FG | 738.03 | *** | 59.32 | *** | 741.40 | *** | 50.65 | *** | 710.83 | *** | 16.41 | * |
| ASC_LC | 711.11 | *** | 77.46 | *** | 716.77 | *** | 69.85 | *** | 673.06 | *** | 27.01 | *** |
| ASC_MG * vr |  |  |  |  | -10.63 |  | 51.50 | *** | -2.76 |  | 33.65 | *** |
| ASC_FG * vr |  |  |  |  | -6.97 |  | 46.23 | *** | -5.26 |  | 24.82 | ** |
| ASC_LC * vr |  |  |  |  | -16.73 |  | 56.79 | *** | -9.05 |  | 36.48 | *** |
| ASC_MG * age |  |  |  |  |  |  |  |  | -8.09 | ** | 1.86 | *** |
| ASC_FG * age |  |  |  |  |  |  |  |  | -0.54 |  | 1.79 | *** |
| ASC_LC * age |  |  |  |  |  |  |  |  | -0.35 |  | 1.57 | *** |
| ASC_MG * fem |  |  |  |  |  |  |  |  | 0.21 |  | 5.83 |  |
| ASC_FG * fem |  |  |  |  |  |  |  |  | 18.74 | * | 18.53 | ** |
| ASC_LC * fem |  |  |  |  |  |  |  |  | 32.32 | *** | 4.57 |  |
| ASC_MG * inc |  |  |  |  |  |  |  |  | 3.82 | * | 2.56 |  |
| ASC_FG * inc |  |  |  |  |  |  |  |  | 1.02 |  | 0.19 |  |
| ASC_LC * inc |  |  |  |  |  |  |  |  | 1.14 |  | 2.59 |  |
| ASC_LC * diet |  |  |  |  |  |  |  |  | -6.37 |  | 0.91 |  |
| ASC_LC * swt |  |  |  |  |  |  |  |  | -15.70 |  | 25.65 | ** |
| ASC_FG * col |  |  |  |  |  |  |  |  | 5.28 |  | 5.98 |  |
| ASC_LC * col |  |  |  |  |  |  |  |  | 12.34 |  | 11.83 |  |
| ASC_FG * df |  |  |  |  |  |  |  |  | 41.24 | *** | 29.82 | *** |
| ASC_LC * df |  |  |  |  |  |  |  |  | 18.81 | * | 40.67 | *** |
| ASC_LC * lc |  |  |  |  |  |  |  |  | 52.17 | *** | 0.07 |  |
| ASC_MG * lp |  |  |  |  |  |  |  |  | 22.58 | * | 6.34 |  |
| ASC_MG * eatmg |  |  |  |  |  |  |  |  | 25.24 | * | 25.16 |  |
| ASC_FG * eatfg |  |  |  |  |  |  |  |  | 9.41 |  | 8.55 |  |
| ASC_LC * eatlc |  |  |  |  |  |  |  |  | 54.50 | *** | 11.52 |  |
| Number of observations | 2,043.00 |  |  |  | 2,043.00 |  |  |  | 2,043.00 |  |  |  |
| Log-Likelihood | -1,817.37 |  |  |  | -1,813.38 |  |  |  | -1,743.99 |  |  |  |
| AIC | 3,648.74 |  |  |  | 3,652.76 |  |  |  | 3,593.98 |  |  |  |
| BIC | 3,688.10 |  |  |  | 3,725.84 |  |  |  | 3,891.95 |  |  |  |

Note:

1) All estimation results are based on the WTP space formulations with fixed price parameters.
2) MG, FG, and LC indicate Mygra, Frugra, and Frugra LC, respectively.
3) Values are MWTP per bag of each RTEC commodity. Because the weights per bag of Mygra, Frugra, and Frugra LC were 700 g, 750 g, and 600 g, respectively, the MWTPs per 100 g were obtained by dividing the values in the table by 7, 7.5, and 6.
4) ***, **, and * indicate significance at the 1%, 5%, and 10% levels, respectively.
