# Supplementary material for: Can immersive virtual reality be an alternative world for eliciting consumer willingness to pay for food?
Source: PLoS One. 2026 Oct 22;21(10):e0361063. doi: 10.1371/journal.pone.0361063 (PMC13690231; doi:10.1371/journal.pone.0361063)
Supplement: Supplementary file 2 [file pone.0361063.s002.docx]

## S2 Table. Discrete choice model estimation and WTP results (preference space)

|  | **Model 1** | | | | | | **Model 2** | | | | | | **Model 3** | | | | | |
| --- | --- | --- | --- | --- | --- | --- | --- | --- | --- | --- | --- | --- | --- | --- | --- | --- | --- | --- |
|  | Mean | | SD | | MWTP | | Mean | | SD | | MWTP | | Mean | | SD | | MWTP | |
| **ASC_MG** | 22.84 | *** | 2.72 | *** | 630.81 | *** | 22.99 | *** | 2.71 | *** | 635.88 | *** | 27.72 | *** | 1.20 | *** | 763.66 | *** |
| **ASC_FG** | 26.68 | *** | 2.15 | *** | 736.70 | *** | 26.81 | *** | 2.01 | *** | 741.64 | *** | 25.61 | *** | 0.12 |  | 705.62 | *** |
| **ASC_LC** | 25.68 | *** | 2.83 | *** | 709.25 | *** | 25.92 | *** | 2.59 | *** | 717.08 | *** | 25.05 | *** | 0.21 |  | 690.21 | *** |
| **Price** | -0.04 | *** |  |  |  |  | -0.04 | *** |  |  |  |  | -0.04 | *** |  |  |  |  |
| **ASC_MG * vr** |  |  |  |  |  |  | -0.41 |  | 0.34 |  | -11.26 |  | -0.14 |  | 0.34 |  | -3.82 |  |
| **ASC_FG * vr** |  |  |  |  |  |  | -0.48 |  | 1.16 | ** | -13.23 |  | -0.42 |  | 0.97 | ** | -11.68 |  |
| **ASC_LC * vr** |  |  |  |  |  |  | -0.64 |  | 1.40 | * | -17.80 |  | -0.29 |  | 1.19 | ** | -8.12 |  |
| **ASC_MG * age** |  |  |  |  |  |  |  |  |  |  |  |  | -0.28 | ** | 0.09 | *** | -7.83 | ** |
| **ASC_FG * age** |  |  |  |  |  |  |  |  |  |  |  |  | -0.01 |  | 0.07 | *** | -0.20 |  |
| **ASC_LC * age** |  |  |  |  |  |  |  |  |  |  |  |  | -0.04 |  | 0.07 | *** | -1.13 |  |
| **ASC_MG * fem** |  |  |  |  |  |  |  |  |  |  |  |  | -0.10 |  | 0.51 |  | -2.70 |  |
| **ASC_FG * fem** |  |  |  |  |  |  |  |  |  |  |  |  | 0.84 | ** | 0.85 | * | 23.17 | ** |
| **ASC_LC * fem** |  |  |  |  |  |  |  |  |  |  |  |  | 1.25 | *** | 0.46 |  | 34.52 | *** |
| **ASC_MG * inc** |  |  |  |  |  |  |  |  |  |  |  |  | 0.14 | * | 0.01 |  | 3.91 | * |
| **ASC_FG * inc** |  |  |  |  |  |  |  |  |  |  |  |  | 0.03 |  | 0.04 |  | 0.82 |  |
| **ASC_LC * inc** |  |  |  |  |  |  |  |  |  |  |  |  | -0.01 |  | 0.16 | *** | -0.16 |  |
| **ASC_LC * diet** |  |  |  |  |  |  |  |  |  |  |  |  | -0.12 |  | 0.17 |  | -3.42 |  |
| **ASC_LC * swt** |  |  |  |  |  |  |  |  |  |  |  |  | -0.47 |  | 0.51 |  | -12.98 |  |
| **ASC_FG * col** |  |  |  |  |  |  |  |  |  |  |  |  | 0.17 |  | 0.14 |  | 4.56 |  |
| **ASC_LC * col** |  |  |  |  |  |  |  |  |  |  |  |  | 0.56 |  | 0.24 |  | 15.32 |  |
| **ASC_FG * df** |  |  |  |  |  |  |  |  |  |  |  |  | 1.42 | *** | 1.08 | ** | 38.99 | *** |
| **ASC_LC * df** |  |  |  |  |  |  |  |  |  |  |  |  | 0.70 | * | 1.85 | *** | 19.25 | * |
| **ASC_LC * lc** |  |  |  |  |  |  |  |  |  |  |  |  | 2.00 | *** | 0.20 |  | 55.08 | *** |
| **ASC_MG * lp** |  |  |  |  |  |  |  |  |  |  |  |  | 0.56 |  | 1.16 | *** | 15.41 |  |
| **ASC_MG * eatmg** |  |  |  |  |  |  |  |  |  |  |  |  | 0.75 |  | 1.16 |  | 20.75 |  |
| **ASC_FG * eatfg** |  |  |  |  |  |  |  |  |  |  |  |  | 0.35 |  | 0.48 |  | 9.76 |  |
| **ASC_LC * eatlc** |  |  |  |  |  |  |  |  |  |  |  |  | 1.86 | *** | 0.10 |  | 51.18 | *** |
| **Number of observations** | 2,043.00 |  |  |  |  |  | 2,043.00 |  |  |  |  |  | 2,043.00 |  |  |  |  |  |
| **Log-likelihood** | -1,818.61 |  |  |  |  |  | -1,817.36 |  |  |  |  |  | -1,746.14 |  |  |  |  |  |
| **AIC** | 3,651.21 |  |  |  |  |  | 3,660.71 |  |  |  |  |  | 3,598.27 |  |  |  |  |  |
| **BIC** | 3,690.57 |  |  |  |  |  | 3,733.80 |  |  |  |  |  | 3,896.25 |  |  |  |  |  |

Note:

1) Mean and SD results are based on the preference space formulations with fixed price parameters. The models in which the price coefficient was assumed to follow either a log-normal or zero-censored normal distribution failed to converge and the corresponding results are therefore not reported.
2) MWTP was calculated as the negative ratio of the non-price coefficient to the corresponding price coefficient. Standard errors are estimated using the Krinsky and Robb parametric bootstrapping method.
3) MG, FG, and LC indicate Mygra, Frugra, and Frugra LC, respectively.
4) MWTP values are per bag of each RTEC commodity. Because the weights per bag of Mygra, Frugra, and Frugra LC were 700 g, 750 g, and 600 g, respectively, the MWTPs per 100 g were obtained by dividing the values in the table by 7, 7.5, and 6.
5) ***, **, and * indicate significance at the 1%, 5%, and 10% levels, respectively.
